# Supplementary figures and images for: Structure of the chromatin remodelling enzyme Chd1 bound to a ubiquitinylated nucleosome
Source: eLife. 2018 Aug 6;7:e35720. doi: 10.7554/eLife.35720 (PMC6118821; doi:10.7554/eLife.35720)

## Slide 1
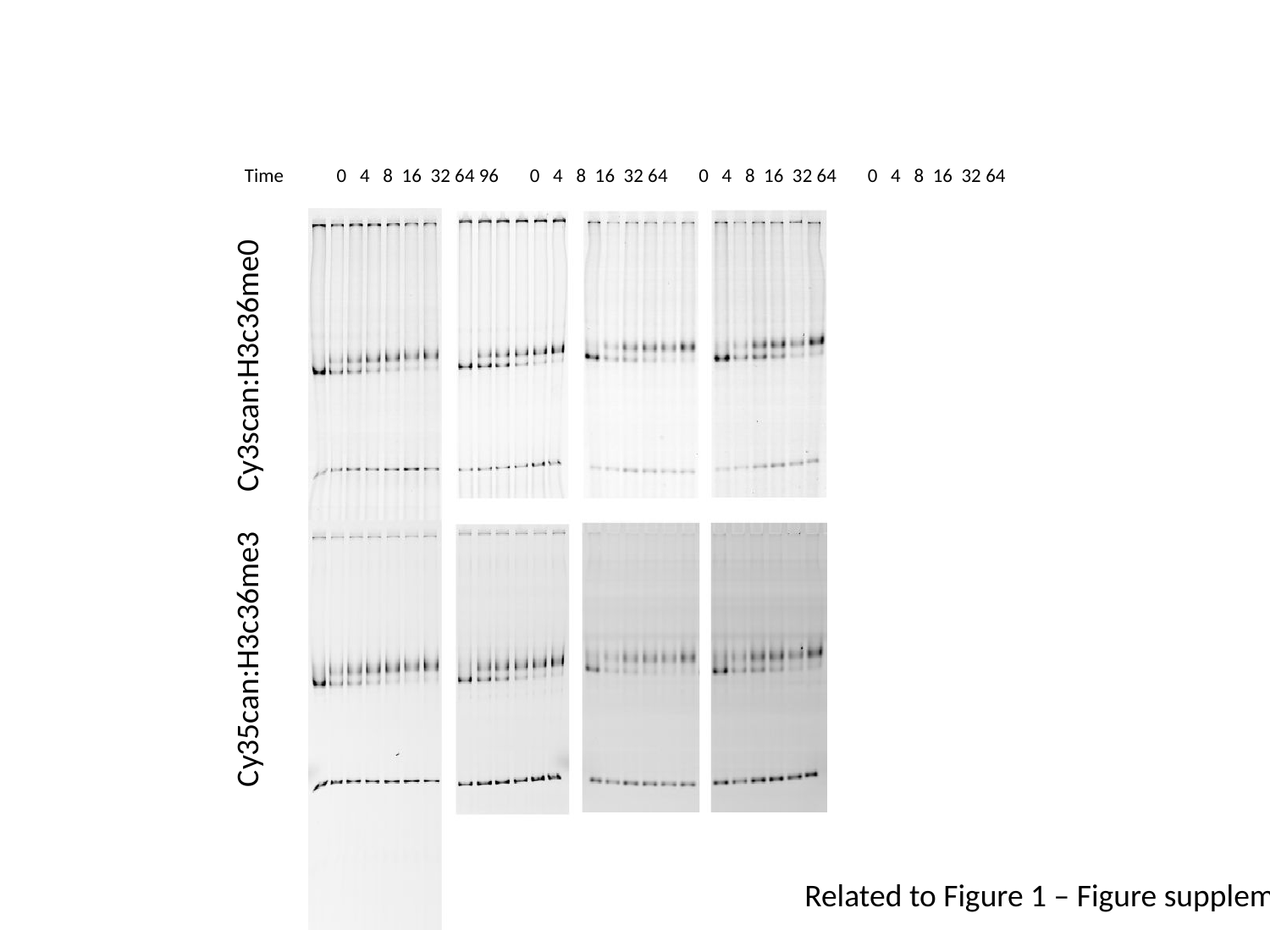

Time 0 4 8 16 32 64 96 0 4 8 16 32 64 0 4 8 16 32 64 0 4 8 16 32 64
#
Cy3scan:H3c36me0
Cy35can:H3c36me3
Related to Figure 1 – Figure supplement 1C

Supplement: Figure 1—source data 1. [file elife-35720-fig1-data1.pptx]

## Slide 1
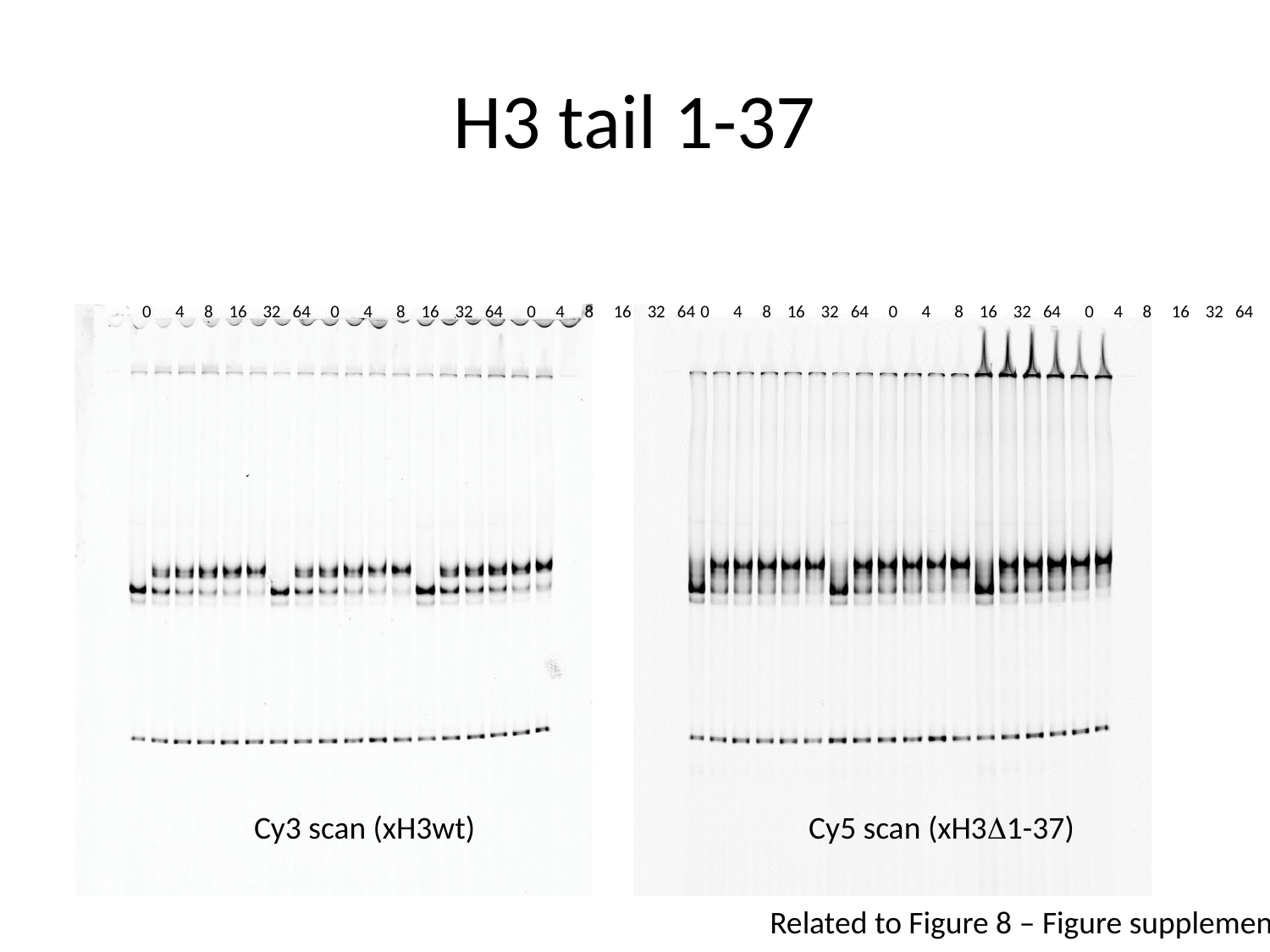

# H3 tail 1-37
0 4 8 16 32 64 0 4 8 16 32 64 0 4 8 16 32 64
0 4 8 16 32 64 0 4 8 16 32 64 0 4 8 16 32 64
Cy3 scan (xH3wt)
Cy5 scan (xH3D1-37)
Related to Figure 8 – Figure supplement 1A

Supplement: Figure 8—source data 1. [file elife-35720-fig8-data1.pptx]

## Slide 1
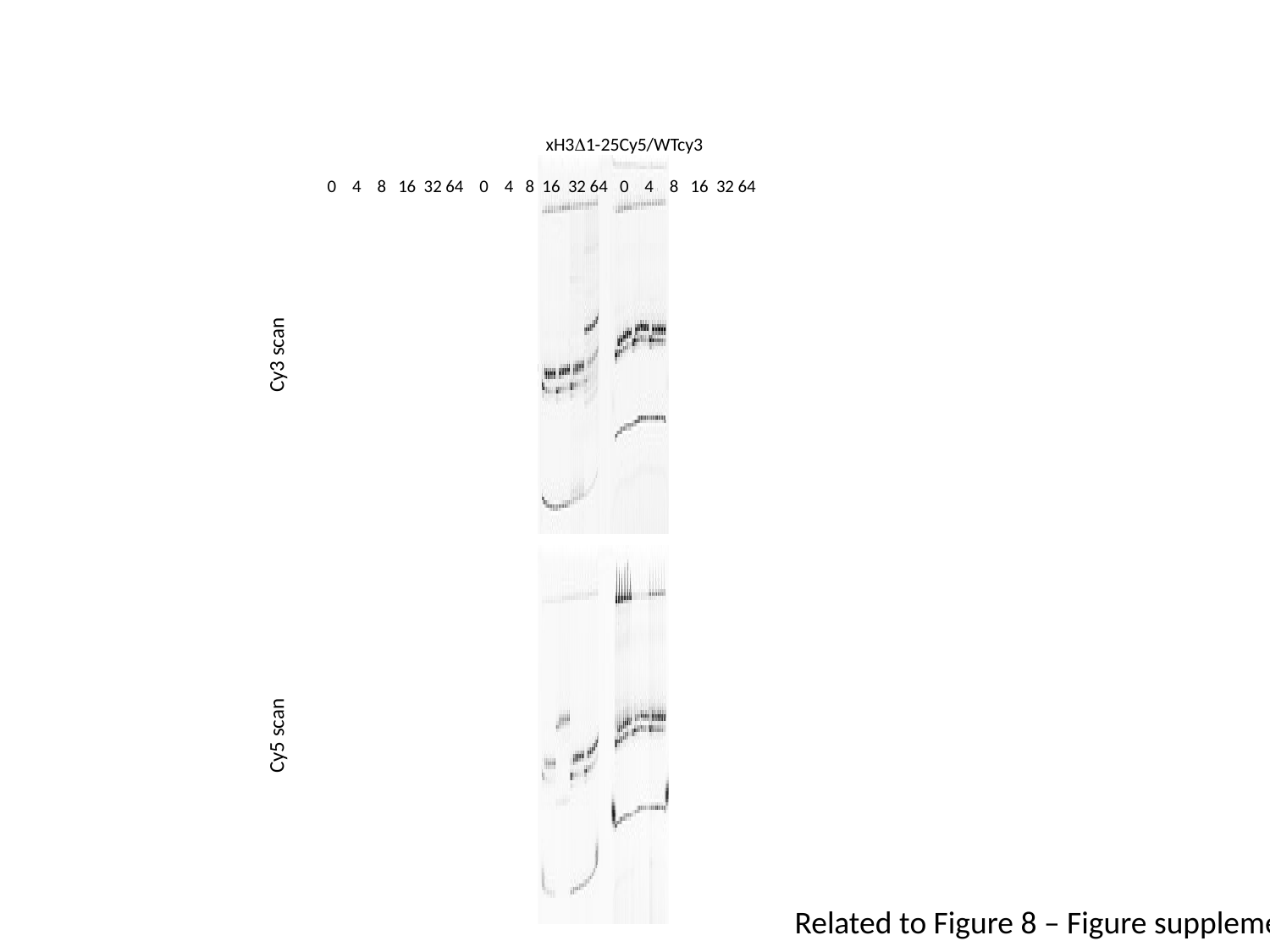

xH3D1-25Cy5/WTcy3
0 4 8 16 32 64 0 4 8 16 32 64 0 4 8 16 32 64
Cy5 scan			Cy3 scan
Related to Figure 8 – Figure supplement 1B

Supplement: Figure 8—source data 4. [file elife-35720-fig8-data4.pptx]
