# Supplementary material for: Structure of the chromatin remodelling enzyme Chd1 bound to a ubiquitinylated nucleosome
Source: eLife. 2018 Aug 6;7:e35720. doi: 10.7554/eLife.35720 (PMC6118821; doi:10.7554/eLife.35720)
Supplement: Figure 2—source data 1. [file elife-35720-fig2-data1.pptx]

## Slide 1
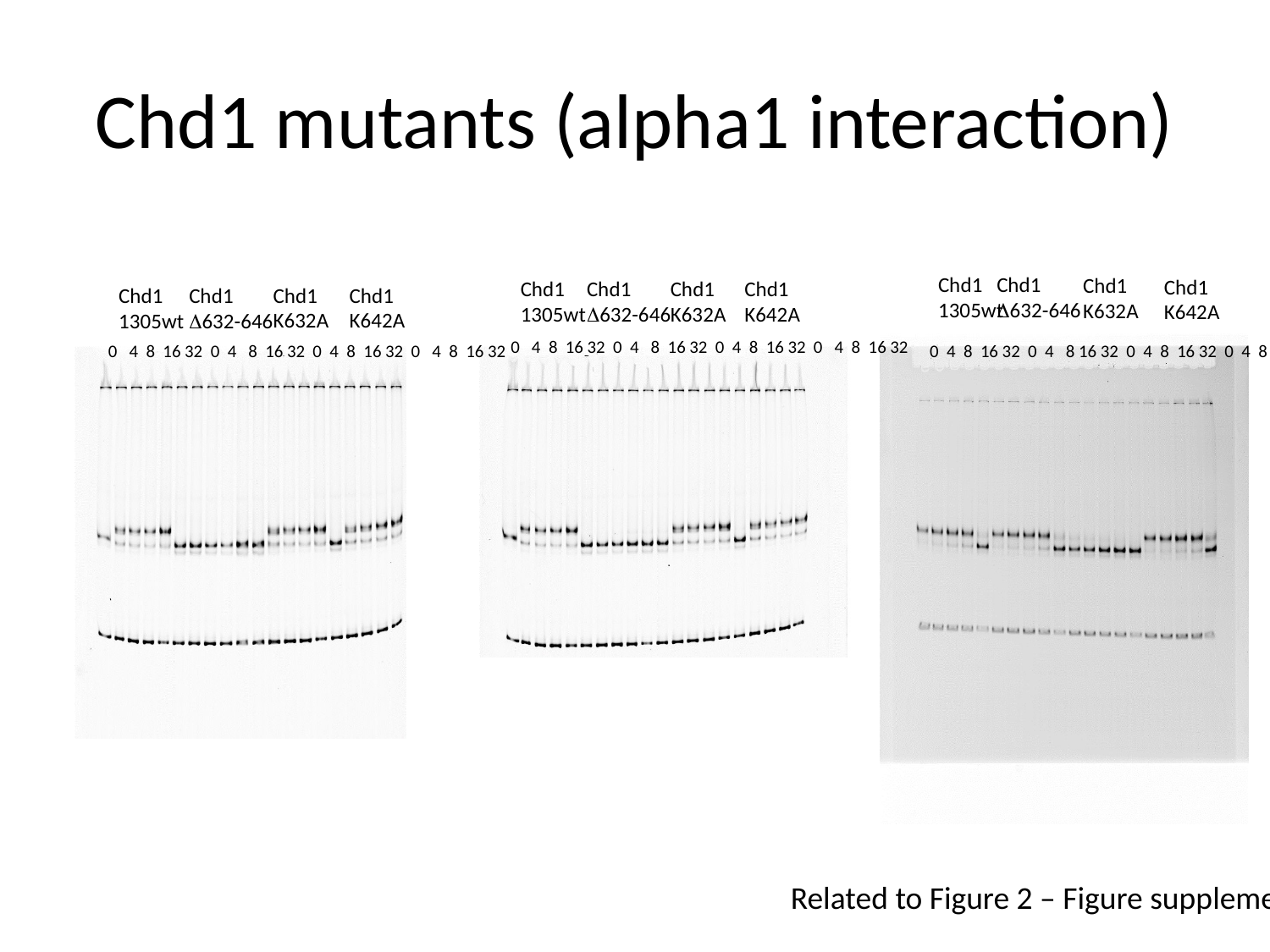

# Chd1 mutants (alpha1 interaction)
Chd1
D632-646
Chd1
1305wt
Chd1
K632A
Chd1
K642A
Chd1
K632A
Chd1
K642A
Chd1
1305wt
Chd1
D632-646
Chd1
K632A
Chd1
K642A
Chd1
1305wt
Chd1
D632-646
0 4 8 16 32 0 4 8 16 32 0 4 8 16 32 0 4 8 16 32
0 4 8 16 32 0 4 8 16 32 0 4 8 16 32 0 4 8 16 32
0 4 8 16 32 0 4 8 16 32 0 4 8 16 32 0 4 8 16 32
Related to Figure 2 – Figure supplement 5A
