# Supplementary material for: Structure of the chromatin remodelling enzyme Chd1 bound to a ubiquitinylated nucleosome
Source: eLife. 2018 Aug 6;7:e35720. doi: 10.7554/eLife.35720 (PMC6118821; doi:10.7554/eLife.35720)
Supplement: Figure 8—source data 3. [file elife-35720-fig8-data3.pptx]

## Slide 1
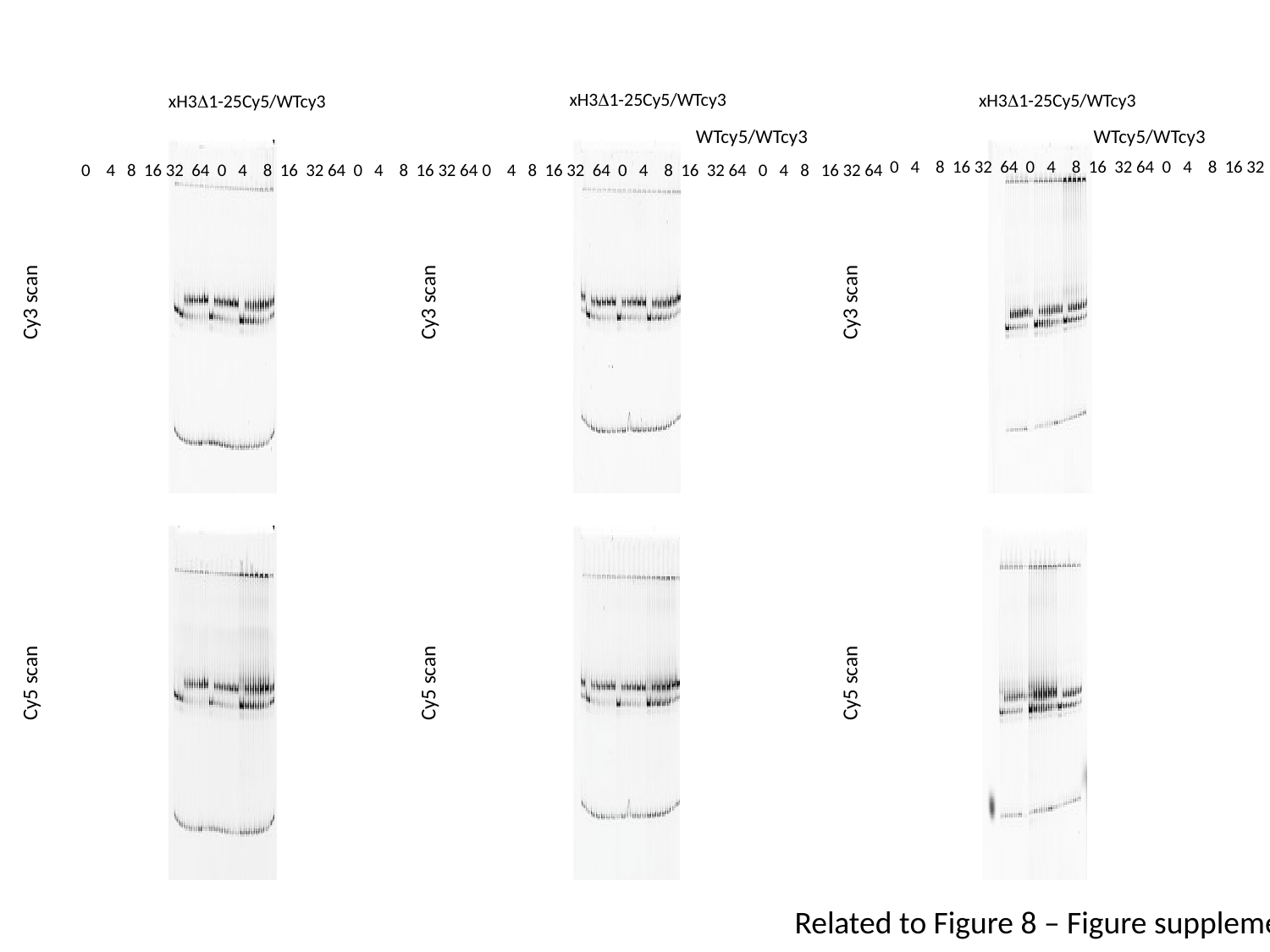

xH3D1-25Cy5/WTcy3
xH3D1-25Cy5/WTcy3
xH3D1-25Cy5/WTcy3
WTcy5/WTcy3
WTcy5/WTcy3
0 4 8 16 32 64 0 4 8 16 32 64 0 4 8 16 32
0 4 8 16 32 64 0 4 8 16 32 64 0 4 8 16 32 64
0 4 8 16 32 64 0 4 8 16 32 64 0 4 8 16 32 64
Cy5 scan			Cy3 scan
Cy5 scan			Cy3 scan
Cy5 scan			Cy3 scan
Related to Figure 8 – Figure supplement 1B
