# Supplementary material for: Structure of the chromatin remodelling enzyme Chd1 bound to a ubiquitinylated nucleosome
Source: eLife. 2018 Aug 6;7:e35720. doi: 10.7554/eLife.35720 (PMC6118821; doi:10.7554/eLife.35720)
Supplement: Figure 8—source data 5. [file elife-35720-fig8-data5.pptx]

## Slide 1
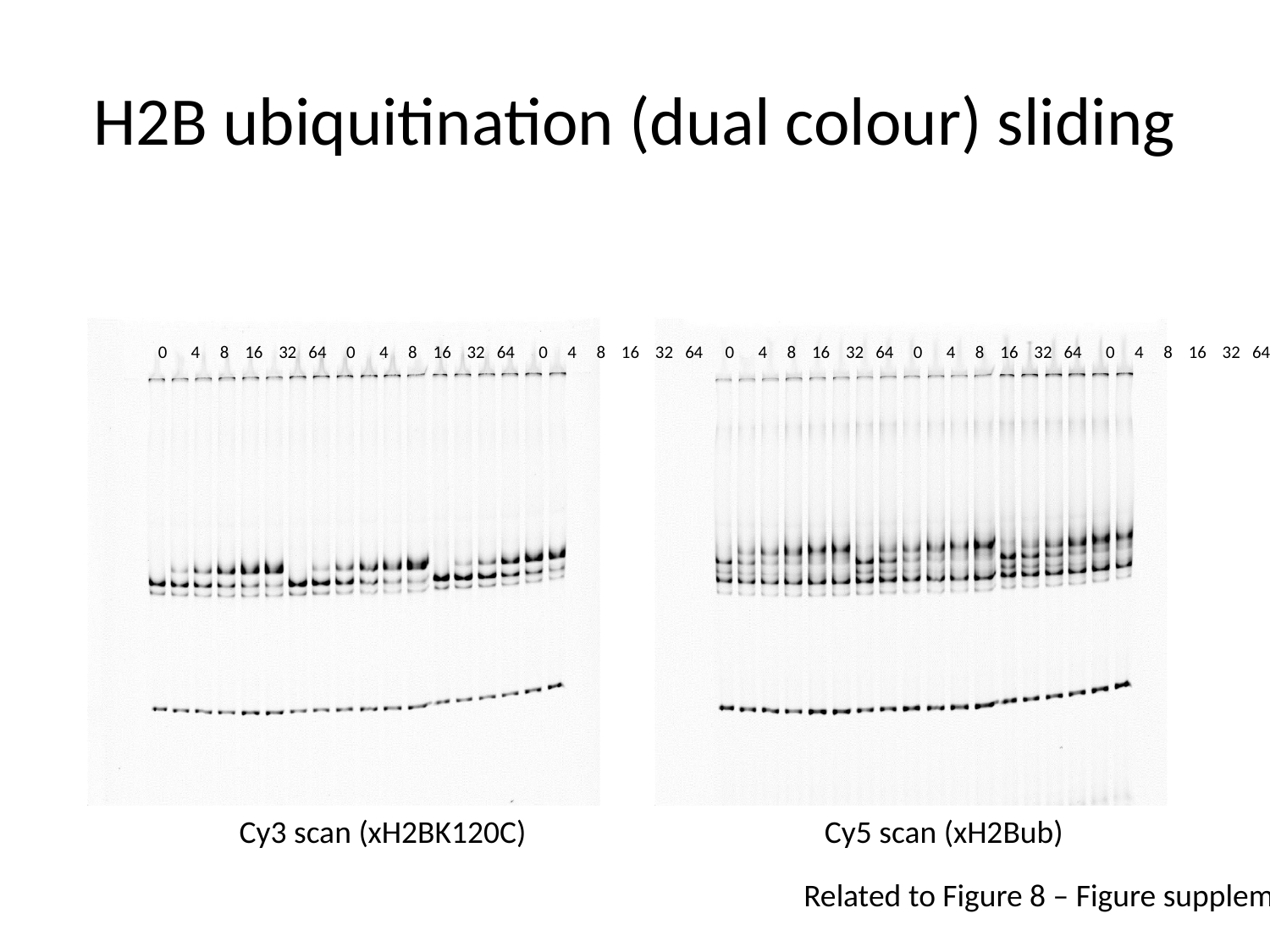

# H2B ubiquitination (dual colour) sliding
0 4 8 16 32 64 0 4 8 16 32 64 0 4 8 16 32 64
0 4 8 16 32 64 0 4 8 16 32 64 0 4 8 16 32 64
Cy3 scan (xH2BK120C)
Cy5 scan (xH2Bub)
Related to Figure 8 – Figure supplement 2
