# Supplementary material for: Structure of the chromatin remodelling enzyme Chd1 bound to a ubiquitinylated nucleosome
Source: eLife. 2018 Aug 6;7:e35720. doi: 10.7554/eLife.35720 (PMC6118821; doi:10.7554/eLife.35720)
Supplement: Supplementary file 1. [file elife-35720-supp1.docx]

Primers used in generating the deletion ∆632-646 on Chd1

Forward 5’ -TCTTTACTGAATATTATGAACGAGTTGAAAAAGGCATCGAAC-3’

Revers 5’ -CAGAATATTTTTATAGTACTCAGTCTGTACGTCGGACAA-3’

DNA sequence of Chd1 ∆632-646

ATGGCAGCCAAGGATATTTCTACTGAAGTGCTGCAAAATCCTGAACTATATGGGCTGAGGCGGTCACATAGAGCTGCTGC

GCATCAACAAAACTACTTCAACGATAGCGATGATGAGGACGATGAAGATAATATTAAACAGTCGAGGAGGAAGAGAATGA

CCACGATTGAAGACGACGAAGACGAATTTGAAGATGAAGAAGGCGAAGAAGATTCTGGAGAGGACGAAGATGAGGAAGAT

TTTGAAGAAGATGATGATTATTATGGCTCTCCTATAAAACAAAATCGATCAAAACCGAAATCAAGAACAAAATCAAAGTC

TAAATCTAAGCCAAAATCTCAATCTGAAAAGCAATCTACAGTGAAAATACCAACAAGGTTTTCTAATCGTCAAAACAAAA

CGGTAAATTATAACATCGATTATAGCGATGATGACCTCTTAGAATCTGAAGATGACTATGGTTCCGAGGAGGCTTTATCA

GAGGAAAATGTGCATGAAGCATCTGCCAATCCTCAACCAGAGGACTTCCACGGTATTGATATTGTTATCAATCACAGACT

AAAGACATCTTTGGAAGAAGGAAAGGTCTTGGAAAAGACTGTTCCTGACCTCAATAACTGCAAGGAAAATTATGAATTCT

TAATCAAATGGACAGATGAATCTCATCTCCACAATACCTGGGAAACTTATGAATCCATTGGCCAAGTCCGTGGATTGAAG

AGGTTGGACAACTATTGTAAGCAATTCATCATCGAAGATCAACAAGTAAGATTAGATCCATACGTTACCGCCGAAGATAT

AGAGATCATGGACATGGAACGTGAACGTAGGCTTGATGAATTTGAGGAGTTCCACGTGCCAGAAAGGATTATTGATAGCC

AACGTGCTTCGTTGGAAGACGGCACCTCACAATTACAGTATTTAGTTAAATGGCGTCGTCTAAATTACGACGAGGCTACT

TGGGAGAATGCCACAGATATCGTGAAATTGGCACCTGAACAAGTGAAACATTTTCAAAACAGAGAAAACTCTAAGATCCT

CCCACAATATTCCAGTAATTATACTTCACAGAGACCGCGTTTTGAGAAGTTAAGCGTGCAACCTCCGTTCATTAAAGGTG

GGGAATTAAGAGATTTTCAACTAACTGGTATTAATTGGATGGCATTTTTGTGGTCCAAAGGTGATAATGGTATACTGGCA

GATGAGATGGGCCTGGGAAAAACGGTCCAGACTGTCGCCTTTATCAGTTGGCTGATATTTGCTCGTAGACAAAACGGACC

TCACATCATTGTCGTTCCTTTATCGACAATGCCTGCCTGGTTGGATACTTTTGAGAAATGGGCGCCTGATTTGAATTGTA

TATGCTATATGGGCAACCAAAAATCAAGAGATACCATTCGAGAATATGAATTTTACACCAATCCAAGGGCAAAAGGGAAA

AAAACAATGAAATTTAATGTTTTATTAACAACATACGAGTACATCTTAAAGGATCGTGCTGAATTAGGAAGTATAAAATG

GCAATTTATGGCCGTTGACGAAGCTCATAGACTAAAAAATGCTGAATCATCCCTTTATGAATCATTAAACAGTTTCAAGG

TCGCCAACCGTATGTTAATCACAGGCACACCTCTTCAGAATAATATTAAAGAGTTAGCTGCGTTGGTTAATTTCCTAATG

CCCGGAAGGTTTACGATTGATCAGGAGATTGATTTTGAAAACCAAGATGAAGAGCAAGAAGAATATATTCATGATTTACA

CCGAAGAATACAGCCTTTTATTCTTCGTCGGTTGAAGAAAGACGTAGAAAAATCACTTCCATCAAAGACAGAGCGTATTT

TAAGAGTTGAATCTTTACTGAATATTATGAACGAGTTGAAAAAGGCATCGAACCATCCATATCTCTTCGATAATGCTGAA

GAGCGCGTCTTACAGAAATTTGGGGATGGTAAAATGACTCGAGAAAACGTACTAAGAGGTTTGATCATGTCTTCGGGTAA

GATGGTTCTTTTAGACCAATTATTGACCAGATTGAAGAAAGATGGGCACCGCGTGTTGATTTTTTCACAAATGGTCAGAA

TGCTTGACATTTTAGGCGACTATTTATCCATTAAAGGTATTAACTTCCAAAGGTTAGATGGTACAGTACCATCTGCTCAA

AGAAGGATATCTATTGATCATTTCAACTCTCCGGATTCAAACGACTTCGTATTTTTACTTTCTACTCGTGCTGGTGGTTT

GGGTATCAACTTAATGACCGCCGATACCGTTGTGATTTTTGATTCCGATTGGAATCCGCAAGCCGATTTACAAGCAATGG

CTAGAGCTCATCGTATTGGCCAAAAAAATCACGTTATGGTGTATAGGTTGGTTTCAAAAGACACAGTAGAGGAAGAGGTA

TTAGAAAGAGCACGGAAGAAAATGATTCTGGAATATGCTATTATTTCTCTTGGAGTGACAGATGGTAACAAATACACTAA

GAAGAATGAACCAAATGCCGGTGAATTATCGGCAATCCTGAAGTTCGGTGCAGGCAATATGTTCACTGCCACAGACAACC

AGAAAAAATTGGAGGATCTTAACTTGGATGATGTTTTGAATCATGCAGAGGATCACGTTACTACTCCAGACTTAGGAGAG

TCGCATCTTGGCGGTGAAGAGTTTTTGAAGCAATTCGAAGTGACTGATTATAAAGCTGATATAGATTGGGATGATATCAT

TCCAGAAGAAGAACTAAAAAAACTCCAAGATGAAGAGCAGAAACGCAAGGATGAAGAATATGTTAAAGAACAACTTGAAA

TGATGAATAGAAGGGATAACGCCCTAAAAAAAATAAAAAACAGTGTTAATGGTGATGGGACCGCCGCAAACTCAGATTCT

GACGACGATAGTACTTCCAGATCTTCTAGAAGAAGAGCAAGAGCTAATGACATGGACTCTATTGGTGAATCGGAGGTGAG

AGCTTTGTATAAAGCTATTTTAAAATTTGGCAACCTAAAGGAGATTTTGGACGAGTTGATTGCAGATGGAACCCTGCCGG

TCAAATCATTTGAAAAATACGGTGAAACTTACGATGAAATGATGGAAGCAGCTAAAGACTGTGTACACGAGGAAGAGAAA

AATAGAAAAGAAATCTTGGAGAAACTTGAGAAGCATGCTACCGCCTATAGAGCAAAGCTAAAAAGCGGTGAAATAAAAGC

AGAGAACCAACCAAAGGATAATCCGTTGACTAGATTATCTTTAAAAAAAAGAGAAAAGAAAGCCGTCCTTTTCAACTTTA

AAGGTGTTAAATCTTTAAACGCCGAATCTTTACTAAGTAGAGTGGAGGATTTAAAGTACTTGAAGAACTTGATAAATTCA

AACTACAAAGATGATCCATTAAAGTTTAGCCTAGGCAACAACACACCTAAGCCTGTACAGAATTGGTCATCTAATTGGAC

GAAAGAGGAAGATGAGAAGCTATTGATTGGTGTTTTCAAATATGGATATGGTTCCTGGACACAAATAAGAGACGATCCAT

TTCTAGGCATTACTGATAAAATATTCTTGAATGAAGTTCACAATCCGGTAGCAAAAAAGTCGGCAAGCTCCTCTGATACA

ACACCAACACCCTCAAAGAAAGGAAAGGGAATAACGGGTTCTTCAAAAAAAGTACCTGGTGCCATTCACTTGGGCAGAAG

AGTTGATTATCTATTATCCTTCTTAAGAGGAGGCCTAAATACAAAGAGTCCCAGTGCTGACATAGGCTCGAAAAAACTCC

CTACTGGTCCTTCCAAAAAGAGACAAAGAAAACCTGCTAATCACAGCAAATCCATGACTCCAGAAATT

Primers used to generate H3D1-25:

Protein sequence of Chd1 ∆632-646

MAAKDISTEVLQNPELYGLRRSHRAAAHQQNYFNDSDDEDDEDNIKQSRRKRMTTIEDDEDEFEDEEGEEDSGEDEDEED

FEEDDDYYGSPIKQNRSKPKSRTKSKSKSKPKSQSEKQSTVKIPTRFSNRQNKTVNYNIDYSDDDLLESEDDYGSEEALS

EENVHEASANPQPEDFHGIDIVINHRLKTSLEEGKVLEKTVPDLNNCKENYEFLIKWTDESHLHNTWETYESIGQVRGLK

RLDNYCKQFIIEDQQVRLDPYVTAEDIEIMDMERERRLDEFEEFHVPERIIDSQRASLEDGTSQLQYLVKWRRLNYDEAT

WENATDIVKLAPEQVKHFQNRENSKILPQYSSNYTSQRPRFEKLSVQPPFIKGGELRDFQLTGINWMAFLWSKGDNGILA

DEMGLGKTVQTVAFISWLIFARRQNGPHIIVVPLSTMPAWLDTFEKWAPDLNCICYMGNQKSRDTIREYEFYTNPRAKGK

KTMKFNVLLTTYEYILKDRAELGSIKWQFMAVDEAHRLKNAESSLYESLNSFKVANRMLITGTPLQNNIKELAALVNFLM

PGRFTIDQEIDFENQDEEQEEYIHDLHRRIQPFILRRLKKDVEKSLPSKTERILRVESLLNIMNELKKASNHPYLFDNAE

ERVLQKFGDGKMTRENVLRGLIMSSGKMVLLDQLLTRLKKDGHRVLIFSQMVRMLDILGDYLSIKGINFQRLDGTVPSAQ

RRISIDHFNSPDSNDFVFLLSTRAGGLGINLMTADTVVIFDSDWNPQADLQAMARAHRIGQKNHVMVYRLVSKDTVEEEV

LERARKKMILEYAIISLGVTDGNKYTKKNEPNAGELSAILKFGAGNMFTATDNQKKLEDLNLDDVLNHAEDHVTTPDLGE

SHLGGEEFLKQFEVTDYKADIDWDDIIPEEELKKLQDEEQKRKDEEYVKEQLEMMNRRDNALKKIKNSVNGDGTAANSDS

DDDSTSRSSRRRARANDMDSIGESEVRALYKAILKFGNLKEILDELIADGTLPVKSFEKYGETYDEMMEAAKDCVHEEEK

NRKEILEKLEKHATAYRAKLKSGEIKAENQPKDNPLTRLSLKKREKKAVLFNFKGVKSLNAESLLSRVEDLKYLKNLINS

NYKDDPLKFSLGNNTPKPVQNWSSNWTKEEDEKLLIGVFKYGYGSWTQIRDDPFLGITDKIFLNEVHNPVAKKSASSSDT

TPTPSKKGKGITGSSKKVPGAIHLGRRVDYLLSFLRGGLNTKSPSADIGSKKLPTGPSKKRQRKPANHSKSMTPEI

xH3S28F: TCCGCTCCTGCTACCGGC
xH3p102D1-26R: CTTCATGGTATATCTCCTTCTTAAAGTT

Protein sequence of H3D1-25:

MKSAPATGGVKKPHRYRPGTVALREIRRYQKSTELLIRKLPFQRLVREIAQDFKTDLRFQSSAVMALQEASEAYLVALFEDTNLCAIHAKRVTIMPKDIQLARRIRGERA

DNA sequence of H3D1-25:

ATGAAGTCCGCTCCTGCTACCGGCGGAGTCAAGAAACCTCACCGTTACCGGCCCGGCACAGTCGCTCTCCGCGAGATCCGCCGCTACCAGAAATCCACCGAGCTGCTCATCCGCAAACTGCCTTTCCAGCGCCTGGTCCGGGAGATCGCTCAGGACTTCAAGACCGACCTGCGCTTCCAGAGCTCGGCCGTTATGGCTCTGCAGGAGGCCAGCGAGGCTTATCTGGTCGCTCTCTTTGAGGACACCAACCTGTGCGCCATCCACGCCAAGAGGGTCACCATCATGCCCAAGGACATCCAGCTGGCCCGCAGAATCCGAGGCGAGAGGGCT

Protein sequence of H3D1-37:

MPHRYRPGTVALREIRRYQKSTELLIRKLPFQRLVREIAQDFKTDLRFQSSAVMALQEASEAYLVALFEDTNLCAIHAKRVTIMPKDIQLARRIRGERA

DNA sequence of H3D1-37:

ATGCCTCACCGTTACCGTCCGGGCACAGTCGCTCTCCGCGAGATCCGCCGCTACCAGAAATCCACCGAGCTGCTCATCCGCAAACTGCCTTTCCAGCGCCTGGTCCGGGAGATCGCTCAGGACTTCAAGACCGACCTGCGCTTCCAGAGCTCGGCCGTTATGGCTCTGCAGGAGGCCAGCGAGGCTTATCTGGTCGCTCTCTTTGAGGACACCAACCTGTGCGCCATCCACGCCAAGAGGGTCACCATCATGCCCAAGGACATCCAGCTGGCCCGCAGAATCCGAGGCGAGAGGGCT
